# Inflammatory cells and remodeling in bronchial biopsies from COPD patients and controls

**DOI:** 10.1371/journal.pone.0326267

**Published:** 2025-06-17

**Authors:** Tomas M. Eagan, Rune Nielsen, Ingvild Haaland, Gunnar R. Husebø, Sverre Lehmann, Jon A. Ward, Susan J. Wilson

**Affiliations:** 1 Department of Thoracic Medicine, Haukeland University Hospital, Bergen, Norway; 2 Department of Clinical Science, Faculty of Medicine, University of Bergen, Bergen, Norway; 3 Histochemistry Research Unit, Faculty of Medicine, University of Southampton, Southampton, United Kingdom; National and Kapodistrian University of Athens, GREECE

## Abstract

**Background:**

The understanding of inflammation and remodeling in the bronchial wall of COPD patients with varying disease severity remains incomplete.

**Methods:**

35 healthy controls and 47 volunteer COPD patients underwent bronchoscopy with bronchoalveolar lavage (BAL) and sampling by endobronchial biopsies in 2014–2015 as part of the MicroCOPD Study. Biopsies were embedded in glycol methyl acrylate (GMA) resin and examined by immunohistochemistry and staining for enumeration of CD3 + , CD4 + , CD8 + , CD20 + , CD68 + , EG2 + , and NE+ inflammatory cells, as well as endothelial cells (EN4) and smooth muscle actin (SMA). Mucus cells were stained by periodic acid-schiff (PAS), and toluidine blue to visualize the reticular basement membrane (RBM).

**Results:**

The numbers of macrophages and eosinophils were higher, and vascularity increased in the submucosa in COPD patients compared with healthy controls. In healthy smokers there were lower numbers of lymphocytes (CD3 +, CD4 +, CD8 +, CD20+) than never smokers. However, COPD patients with GOLD I/II had higher numbers of eosinophils and larger smooth muscle area compared with GOLD III/IV. COPD exacerbations the last year, blood eosinophils, and use of inhaled corticosteroids did not affect levels of inflammation or remodeling.

**Conclusion:**

Smoking alters inflammation in healthy controls, while specific patterns of macrophages, eosinophils, and vascularity distinguish COPD from non-COPD in bronchial biopsies.

## Introduction

Chronic obstructive pulmonary disease (COPD) is a chronic inflammatory condition characterized by fixed airflow obstruction. The disease is highly heterogeneous, with considerable variation among patients in manifestations such as emphysema and chronic bronchitis [1]. While some COPD patients experience frequent exacerbations [2], often triggered by infections [3], others have none.

The primary cause of COPD is prolonged exposure to inhaled noxious agents, most often tobacco smoke. However, the exact pathogenesis remains unclear. Fixed airflow obstruction results from a combination of small airway collapse due to the loss of elastic support tissue and emphysema, as well as airway inflammation leading to edema and narrowing of the lumen. Despite this, anti-inflammatory therapies such as inhaled corticosteroids (ICS) have shown limited effectiveness in COPD patients [4,5]. This has changed recently to some degree with the realization that COPD patients with higher blood eosinophil counts may benefit from ICS [6,7].

In asthma, both inflammation and airway remodeling are well-established phenomena [8], with substantial evidence supporting the effectiveness of ICS. However, in COPD, fewer studies have examined inflammatory cells in bronchial biopsies compared to healthy controls [9–17]. Across nine key studies, only 205 COPD patients and 213 non-COPD have been included, often with differences in age matching [9–11,13,17], methodologies, and handling of ICS use. Few studies have been powered to look at COPD subgroups, although some studies have specifically addressed the important question of the impact of ICSs [17–20]. Only three studies have attempted to examine differences in COPD characteristics, such as GOLD stage [14], chronic bronchitis [21] and exacerbation frequency [22].

Evidence on airway remodeling in COPD is even more limited. Only three studies have compared the reticular basement membrane (RBM) thickness [22–24], two have compared smooth muscle area [25,26], two have examined goblet cell density [22,27], and two have compared degree of vascularity [24,28]. Furthermore, the effect of smoking in subjects without known lung disease is underexplored, where only four previous studies have specifically compared healthy smokers with healthy never smokers [10,11,15,16].

Overall, there is a significant gap in understanding histopathological changes in medium to large airways in COPD, particularly regarding remodeling and its relationship with local inflammation. Differences among COPD subgroups and the effects of smoking on bronchial inflammation in healthy individuals are also poorly understood.

Here, we present data from the largest bronchoscopy study to date involving both COPD patients and healthy volunteers. Bronchial biopsies were analyzed using glycol methyl acrylate (GMA) resin embedding to produce ultrathin sections for immunohistochemistry. The study aimed to compare airway inflammation and remodeling between COPD patients and healthy controls, explore differences within COPD subgroups, and assess the effects of smoking among healthy adults categorized as current, former, or never-smokers.

## Methods

### Study population

The study population is a subsample from the MicroCOPD Study. Briefly, the MicroCOPD study included 130 COPD patients and 103 healthy controls from Western Norway, with bronchoscopy performed to sample the lower airway microbiome. Data collection started 11th of Aril 2014 and was concluded 5th of June 2015 at the outpatient clinic of the Department of Thoracic Medicine, Haukeland University Hospital, Bergen, Norway. The study was approved by the regional ethical committee (REK Vest case # 2011/1307), all participants provided written consent, and the study was conducted in accordance with the Declaration of Helsinki.

All subjects were examined by a study physician and a detailed medical history including smoking habits, medications, comorbidities, respiratory symptoms, and COPD exacerbations was obtained. Spirometry was performed after inhalation of 0.4 mg salbutamol prior to the bronchoscopy, and the details of the bronchoscopic sampling procedure have been published [29]. Bronchoscopy was performed with oral access while the patient was supine, under local lidocaine anesthesia and optional intravenous alfentanil. Bronchoalveolar lavage (BAL) was collected from the middle lobe, and sterile brushes were used to sample both lungs. Beginning April 28th, 2014, bronchial biopsies were also collected during bronchoscopy using 1.8 mm disposable cupped forceps (Olympus). Three medium-large biopsies were sampled from the fourth to second carina of the right lower lobe, with 5 mL of 0.1 mg/mL adrenaline instilled prior to sampling to minimize bleeding. Due to staffing limitations, biopsies were processed intermittently. This study includes 35 lung-healthy controls and 47 COPD patients with acceptable biopsy specimens for immunohistochemistry.

### Immunohistochemistry

Biopsies were fixed in acetone, embedded in GMA, and sectioned at 2 µm thickness, as described by Britten *et al.* [30]. Only participants with biopsies containing at least 0.49 mm² submucosal area were included [31]. Immunohistochemistry targeted endothelial cells (EN4), smooth muscle actin (SMA), and inflammatory cells: neutrophils (NE), T-cells (CD3, CD4, CD8), B-cells (CD20), macrophages (CD68), and eosinophils (EG2), following the protocol by Collins *et al.* [32].

For measurements of mucus in the epithelium, only participants whose biopsies revealed intact epithelium not tangentially cut, and whose total length was a minimum of 0.1 mm were included [31]. One 2 µm thin section per biopsy was stained with periodic acid-schiff (PAS), and the same cutting procedure and inclusion criteria were used to stain sections with toluidine blue to measure the thickness of the lamina reticularis (RBM).

Biopsy sections from one participant per experiment were used as negative controls, and sections from nasal polyps and tonsils were used as positive controls. All sections were converted to digital images by a Hamamatsu NanoZoomer XR scanner and Aperio ImageScope (version 12.1) was used for image analyses.

### Image analysis

**Fig 1:**
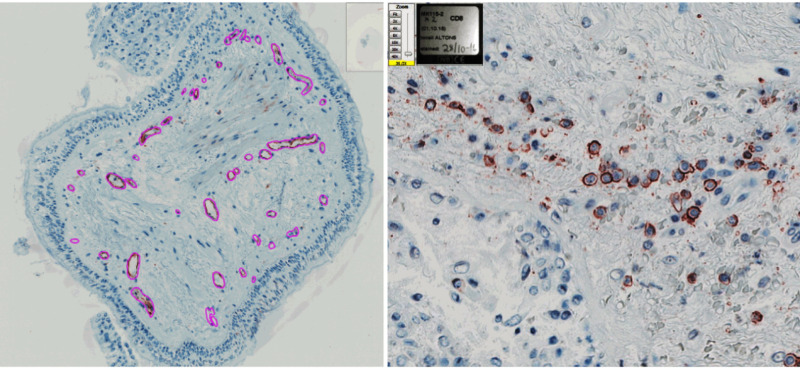
Immunohistochemistry staining of bronchial biopsies showing stained vessels cells (EN4+) on the left and stained T-lymphocytes (CD8+) on the right.

T-cells, B-cells, macrophages, and eosinophils were manually counted and reported as positive cells/mm² of submucosa, excluding muscle and vessels. Neutrophils and epithelial mucus fraction were measured using a positive pixel count algorithm. The thickness of the lamina reticularis was measured as the total area of lamina reticularis/ length of µm epithelium beneath. Muscle fraction was calculated as SMA stained area/mm2 in the submucosa and vascularity was calculated as area fraction of EN4 stained cells/mm^2^ in the submucosa. Fig 1 shows an example of the drawn area for vessels (left), and CD8 + staining cells (right). All histochemistry experiments and image analyses were performed by the same technician, blinded to participant diagnosis.

### Statistical analysis

The statistical analyses were performed with Stata 14.2 and Prism 10.4. A post-hoc power analysis of the examined associations between inflammatory cells and remodeling indices was performed with the program G power [33]. Most indices examined showed non-normal distributions. Kolmogorov-Smirnov tests were used to compare distributions and Kruskal-Wallis tests to compare medians within groups and subgroups. All pairwise correlation coefficients between the inflammation and remodeling variables were calculated by Spearman’s nonparametric rank correlation tests. P values < 0.05 were considered statistically significant.

## Results

The characteristics of the study population are shown in Table 1. Sex and age were comparable between lung-healthy controls and COPD patients. However, smoking status differed significantly, as only controls included never smokers. Slightly more than one-third of the COPD patients had an FEV_1_ < 50% of the predicted value, and more than half used inhaled corticosteroids (Table 1).

**Table 1:** Characteristics of the study population.

	Healthy controls	COPD patients	p^*^
	n = 35	n = 47	
*Sex*			0.34
Men, n (%)	21 (60.0)	33 (70.2)	
*Age*			0.87
Mean (SD)	66.9 (6.6)	67.1 (5.7)	
*Smoking*			<0.01
Never, n (%)	10 (28.6)	0	
Ex, n (%)	17 (48.6)	31 (66.0)	
Current, n (%)	8 (22.9)	16 (34.0)	
*Pack-years smoked* ^**^			0.02
Mean (SD)	23.5 (13.4)	33.7 (18.0)	
*GOLD stage*			
I/II, n (%)	–	30 (63.9)	
III/IV, n (%)	–	17 (36.1)	
*FEV1/FVC ratio*			
Mean (SD)	–	0.46 (0.12)	
*CAT score*			
Median (IQR)	–	16 (10-22)	
*Exacerbations the last 12 months*			
≥1, n (%)	–	14 (29.8)	
*Daily use of inhaled steroids*			
Yes, n (%)	–	29 (61.7)	

* Chi-square test for sex, t-test for age and pack-years, Fishers exact test for smoking

** Among ex or current smokers

The correlations between the inflammatory cells and indices of remodeling in the bronchial biopsies are shown in Tables 2 and 3, for controls and COPD patients respectively.

**Table 2:** Statistically significant correlations^*^ between inflammation and remodeling in bronchial biopsies from healthy controls.

	CD4	CD8	CD20	CD68	EG2	NE	RBM	PAS	SMA	EN4
T-lymphocytes (CD4)	1									
T-lymphocytes (CD8)	0.50	1								
B-lymphocytes (CD20)	0.52	0.52	1							
Macrophages (CD68)	0.44	0.65	0.38	1						
Eosinophils (EG2)					1					
Neutrophils (NE)						1				
Lamina Reticularis (RBM)							1			
Goblet cells (PAS)								1		
Smooth muscle area (SMA)	−0.50								1	
Microvessels (EN4)	0.45	0.52	0.35							1

* Spearman rank correlation coefficients, significance level p=0.05

**Table 3:** Statistically significant correlations^*^ between inflammation and remodeling in bronchial biopsies from COPD patients.

	CD4	CD8	CD20	CD68	EG2	NE	RBM	PAS	SMA	EN4
T-lymphocytes (CD4)	1									
T-lymphocytes (CD8)	0.73	1								
B-lymphocytes (CD20)	0.40	0.41	1							
Macrophages (CD68)	0.73	0.75		1						
Eosinophils (EG2)	0.53	0.59		0.65	1					
Neutrophils (NE)	0.45	0.49		0.56	0.42	1				
Lamina Reticularis (RBM)		0.58		0.55			1			
Goblet cells (PAS)				0.59				1		
Smooth muscle area (SMA)									1	
Microvessels (EN4)	0.56	0.59		0.79	0.56	0.57				1

* Spearman rank correlation coefficients, significance level p=0.05

The numbers represent the Spearman´s nonparametric correlation coefficients, and only statistically significant correlations are shown. In the control group we detected a positive correlation between lymphocytes and macrophages, and between lymphocytes and area fraction of microvessels. In contrast, in the COPD group we found more correlations between nearly all the inflammatory cell types, and between the inflammatory cells and microvessel area fraction. Especially macrophages in COPD patients correlated significantly with nearly all the tested markers of inflammation and remodeling, besides the muscle area fraction (Table 3) .

**Fig 2:**
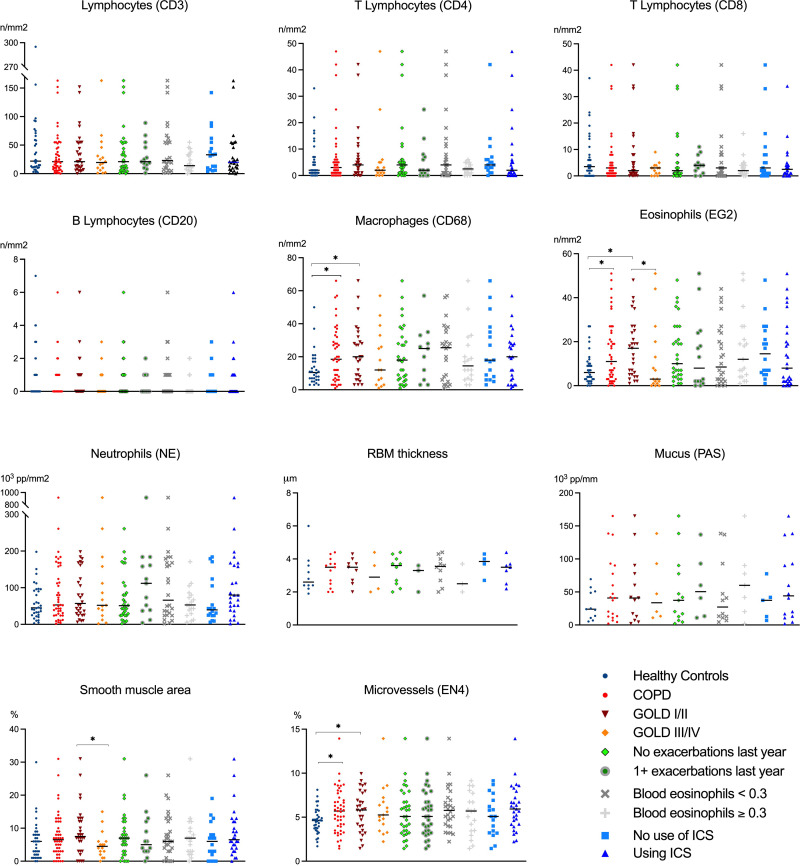
Comparisons of inflammatory markers and signs of remodeling in bronchial biopsies from healthy controls and patients with COPD.

The 11 measures of inflammation and remodeling in the bronchial biopsies are depicted in Fig 2.

The graphs both show comparisons between healthy controls and all COPD patients and COPD patients in GOLD stages I/II and GOLD III/IV, as well as comparisons among the COPD patients of the COPD stages, exacerbations in the previous year, blood eosinophils ≥0.3 * 10^9/L, and use of ICS. Compared with healthy controls, COPD patients had more macrophages and eosinophils, as well as larger areas of microvessels in the submucosa. This was driven entirely by COPD patients with GOLD stages I/II. In fact, COPD patients with GOLD stages III/IV had significantly lower numbers of eosinophils and smaller areas of smooth muscle than did COPD patients with GOLD stages I/II. For all other comparisons no significant differences were found (Fig 2). The medians, interquartile ranges, and p values are provided in S1 and S2 Tables, as are comparisons of differential cell counts in BAL fluid. In addition, the results of the post-hoc power analyses are shown in S1 Table. Predictably, COPD patients had greater numbers of neutrophils in BAL fluid than healthy controls did, but no differences were detected in terms COPD characteristics, much the same as in the bronchial biopsies (S2 Table).

**Fig 3:**
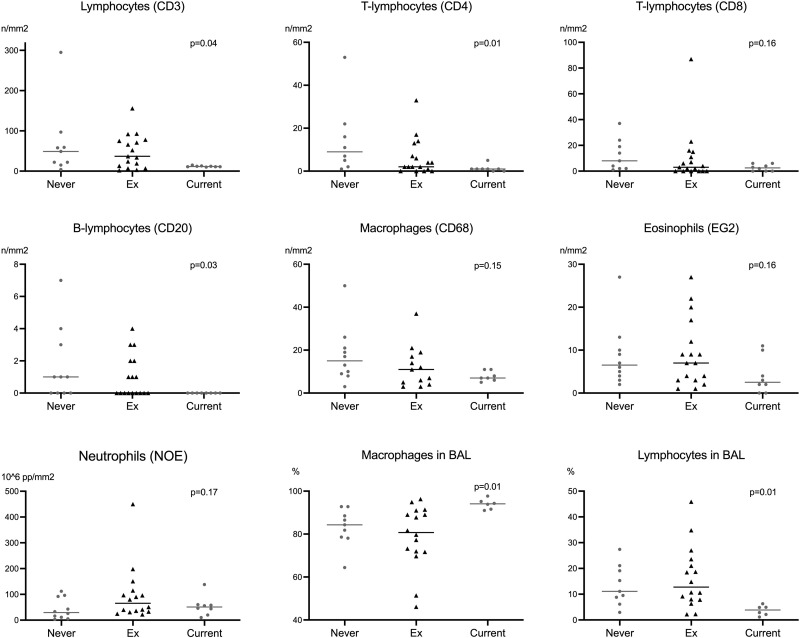
Comparison of inflammatory cells in bronchial biopsies and bronchoalveolar lavage in healthy controls by smoking status.

In terms of the effect of smoking in healthy controls, we found that current smokers had lower numbers of all lymphocyte cell types in bronchial biopsies (Fig 3).

This was also observed in the BAL fluid, where current smokers had lower percentages of lymphocytes and higher percentages of macrophages than never smokers and ex-smokers did. For the remodeling indices, the sample size did not really allow comparisons of the three smoking groups of healthy controls.

However, a comparison is presented between COPD ex and current smokers in S3 Table. The same pattern was observed in BAL fluid with a higher percentage of macrophages and lower percentage of lymphocytes in current smokers with COPD. Furthermore, bronchial biopsies from currently smoking COPD patients had three times more mucus cells than those from ex-smoking COPD patients. For all other comparisons no certain differences were detected in COPD patients between ex-smokers and current smokers (S3 Table).

## Discussion

This study provides valuable insights into the inflammatory and structural features of COPD in bronchial biopsies. Increased vascularity and higher numbers of macrophages and eosinophils were evident in COPD patients compared to healthy controls, while other inflammatory cell populations, including lymphocyte subtypes and neutrophils, showed no clear differences. Interestingly, we observed reduced smooth muscle area and eosinophil counts in advanced (GOLD stage III/IV) COPD compared to milder (GOLD stage I/II) disease. These findings suggest evolving inflammatory and remodeling profiles with disease progression. Smoking suppressed lymphocyte numbers in both BAL and bronchial tissue, affecting COPD patients and controls alike.

Bronchoscopic biopsy studies have yielded conflicting results. One study found significantly lower number of CD4 + cells in bronchial biopsies from COPD patients compared with controls [9], whereas our study and four other studies [10,13,14,17] found no difference. For CD8 + cells, two studies reported higher numbers in COPD [10,17], one lower numbers [14], and ours and two others no difference [9,13]. In the two Swedish studies specifically examining the epithelial lymphocytes, increased numbers of CD8+ [15,16] and CD4+ [15] cells were found in COPD patients. For CD68 + cells, three studies [11,17,34] reported higher numbers in COPD patients similar to our study, whereas one study reported lower numbers [9], and two no difference [10,13]. Notably, Eapen *et al.* [9] categorized CD68 + cells into macrophages and myofibroblasts based on visual appearance, an approach unused in other studies. For neutrophils, most studies reported no difference, though one reported higher numbers [11] and one lower numbers [9]. Finally, for those studies that examined eosinophils, two [11,34] found higher numbers in COPD patients like in our study, however the other three found no difference [10,13,17].

The abovementioned studies vary in methodology. COPD patients were older than controls in most studies, and few studies included the most severely affected patients (GOLD stage IV). Biopsy handling varied, with section thickness ranging from 2–6 µm. Different antibodies were used for staining, and some used number of cells, other areas, to assess cellularity. Observer blinding and automation also varied.

Tobacco smoking and use of inhaled corticosteroids (ICSs) are two factors likely to influence cellularity of inflammatory cells in the bronchial wall and have been analyzed in different ways previously. Some studies compare patients only to smoking controls [13,14,34], others only to non-smoking controls [17], and others have two control groups [9–11,15,16]. In our study, we found an apparent suppressive effect of tobacco smoking on lymphocytes, both in BAL fluid and bronchial biopsies. However, BAL cellularity is compositional data, meaning fewer BAL lymphocytes may reflect an increase in airway macrophages. In bronchial biopsies, our findings supported smoking’s suppressive effect on lymphocytes in non-COPD subjects. Other bronchoscopy studies comparing lymphocytes in healthy smokers and nonsmokers showed no difference [10,11,15], although Roos-Engstrand *et al.* reported higher number of CD3 + cells in the epithelial compartment in healthy smokers than in never smokers [16]. Sample sizes were small in all studies. One previous study examined cellularity before and 24 hours after smoking three cigarettes, without finding differences [35].

One would assume use of ICS might suppress inflammation in the airways, seen as a reduction in immune cells present. Most previous studies on cellularity excluded ICS users [9–11,13], albeit for varying lengths of pre-study time, and some studies split the analyses into patients with and without ICS. Excluding patients on ICS introduces selection bias, whereas splitting samples reduces statistical power. Some studies have specifically examined the effects of ICS on cellularity in bronchial biopsies in from COPD patients [17,19,20,36,37]. In the GLUCOLD randomized controlled trial from the Netherlands it was shown that fluticasone suppressed number of lymphocytes (CD3 +, CD4 +, CD8+) and mast cells in bronchial biopsies [38]. Two later studies on the same cohort showed this effect to be true both in current smokers and ex-smokers [36], and importantly that cellularity increased in subjects who stopped taking fluticasone [37]. In the study by Zanini *et al.*, there were fewer CD68+ and CD8 + cells in bronchial biopsies in COPD patients using ICS compared to patients without ICS, and also vascularity, assessed with vascular endothelial growth factor positive (VEGF+) cells, was decreased in ICS users [17]. The latter finding contrasts with the findings of a longitudinal study by Soltani *et al.*, where several angiogenic growth factors were assessed before and six months after the use of fluticasone or placebo in 23 and 11 COPD patients respectively, and no suppression was found from ICS use [19].

Few studies report airway remodeling in COPD beyond vascularity, and those who do have mostly looked at single aspects and report positive results. Two studies reported a thickening of the RBM [22,23], and one reported increased fragmentation and vascularity within the RBM [39]. Two studies indicated increased numbers of smooth muscle cells [25] or a larger area containing smooth muscle in the bronchial wall of COPD patients [26]. Finally, two studies examined mucus cell volume in the epithelium, and found larger volumes in COPD patients and in non-COPD smokers compared with never-smokers [22,27].

Very few studies have examined the immune cell counts or remodeling by COPD phenotypes. Higham *et al.* reported increased RBM thickness in frequent exacerbators, and a trend toward increased thickness with increasing GOLD stage [22]. Di Stefano *et al.* reported higher numbers of CD3+ and CD8 + cells in patients with severe COPD than in those with mild or moderate COPD [14].

However, in severe COPD, increasing emphysema may reduce total cell counts, as shown by Eapen *et al*. [9]. Loss of lung tissue is an established part of the pathophysiology in COPD, where chronic inflammation, a proteases-antiprotease imbalance, and oxidative stress have been implicated in apoptosis and necrosis [40,41]. In addition, with altered signaling or stem cell depletion impaired tissue repair may be a factor. In more advanced disease there is a larger loss of lung tissue, which may help explain the finding in our study of a smaller smooth muscle area in biopsies from GOLD III/IV patients compared with GOLD I/II.

Although not statistically significant, COPD patients with one or more COPD exacerbations the last year had higher numbers of neutrophils, macrophages, and mucus cells in their biopsies, as one could expect as signs of a persistent immune responses to bacteria. Exacerbations within one year is a very crude measurement, as some exacerbations may be 11 months prior to the study, other 4 months, and the time effects are likely to differ. COPD exacerbations are known to lead to worse prognosis [42] and faster lung function decline [43]. Although not significant, the cellular trend in our biopsies may be worthwhile to explore further in future studies, as a larger study may provide evidence for persistent inflammation induced by COPD exacerbations.

There are some methodological issues to discuss. First, the post-hoc power analyses confirmed that our sample size was too low to provide the necessary power for several associations examined, thus there is a chance of type II errors. Power will vary by the association in question, and was adequate for comparing blood and BAL findings, and number of macrophages in the biopsies for instance, but based on our findings we would suggest an ideal sample size would consist of 100 subjects in each study arm to account for sub-group analyses as well. In an interesting analyses of biopsies from 51 COPD patients sampled twice 10 weeks apart, a sample size of more than 30 would be necessary in each group to reliably predict a doubling or halving of the number of CD8 + cells [44]. Analyses on change is different from cross-sectional data, but the study serves as a reminder that most previous studies are possibly underpowered, at least for subgroup analyses. Second, our study is cross-sectional and can therefore not assess whether inflammation is a precursor or effect of COPD, and therefore also not mechanisms by which inflammation potentially would lead to COPD, or specific COPD phenotypes. Third, bronchial biopsies were taken from the 4–6 carinae in the right lower lobe, which may not be representative of the smaller airways. COPD has significant disease related to the small airways and alveoli, and the effect and/or presence of inflammation in the smaller airways could be different from what we see in the somewhat larger airways.

Despite uncertainties, our study supports an overall trend; increased macrophages and CD8 + lymphocytes in COPD submucosa, with neutrophils more prominent in BAL fluid. Eosinophils may also be increased. The increase in immune cells is more evident in mild-to-moderate COPD, possibly due to disease-stage differences, ICS effects, or overall cellular decline in advanced disease. Increased microvascularity may indicate enhanced leukocyte transport or adaptation to chronic hypoxia.

To better understand the drivers of airway inflammation and remodeling in COPD, integrating bronchial biopsy findings with microbiome data is crucial. Immune responses are closely tied to microbial dysbiosis, which may influence disease progression and heterogeneity. Longitudinal studies with larger, well-characterized cohorts are needed to clarify the interplay between inflammation, remodeling, and clinical phenotypes.

This study highlights increased vascularity and higher numbers of macrophages and eosinophils in COPD bronchial submucosa compared to controls. Smoking exerted a suppressive effect on lymphocyte numbers, while advanced COPD was associated with reduced smooth muscle area. Despite methodological challenges and sample size limitations, our findings contribute to a growing body of evidence suggesting nuanced and evolving inflammatory and remodeling profiles in COPD. Future studies should aim to unravel the complex interdependencies between immune responses, airway structure, and the lung microbiome in this heterogeneous disease.

## Supporting information

S1 Table Indices of inflammation and remodeling in COPD patients and healthy controls. (XLSX)

S2 Table Indices of inflammation and remodeling in COPD patients by GOLD stage, exacerbation frequency, blood eosinophilia, and use of inhaled steroids. (XLSX)

S3 Table Indices of inflammation and remodeling in COPD patients by smoking status. (XLSX)
